# Activating the STING pathway to prevent dormant metastasis in lung adenocarcinoma

**DOI:** 10.1002/mco2.453

**Published:** 2023-12-13

**Authors:** Jin Ma, Xiaoqi Lian, Sijia Liu

**Affiliations:** ^1^ GBA Center for Medical Device Evaluation and Inspection National Medical Products Administration Shenzhen China; ^2^ Dalian Institute of Chemical Physics Chinese Academy of Sciences Dalian China; ^3^ International Biomed‐X Research Center The Second Affiliated Hospital of Zhejiang University School of Medicine, Zhejiang University Hangzhou China

1

In a recent publication in *Nature*, Hu et al. have revealed the potential modulation of stimulators of interferon genes (STING) in preventing the burst of dormant metastasis during the development of lung adenocarcinoma (LUAD).^1^ The dynamic changes in STING activation during the transition of cancer cells from quiescent to reawakened states exert an influence on immune surveillance. This study elucidated the role of STING as a novel checkpoint that effectively prevents the progression of metastasis originating from dormant cancer cells, thereby providing a promising therapeutic avenue for forestalling disease recurrence.

Disseminated tumor cells (DTCs) infiltrating various organs are recognized as a critical source of future metastasis outbreaks, even following a favorable prognosis for the primary cancer lasting from months to decades.^2^ The stationary property enables dormant cancer cells to remain inconspicuous from active cancer cells and escape the treatment. Conceptually, eliminating dormant cancer cells while they are “asleep” would be the ideal way to prevent irreversible overt metastasis. However, the mechanisms of single dormant cancer cells awakening up and departing from their quiescent niches are complicated and tightly regulated by the sophisticated immune microenvironment. Recently, Hu et al. identified the STING pathway plays a pivotal role in modulating the interaction between the metastatic tumor seeds and its surrounding immune surveillance (Figure 1).

**FIGURE 1:**
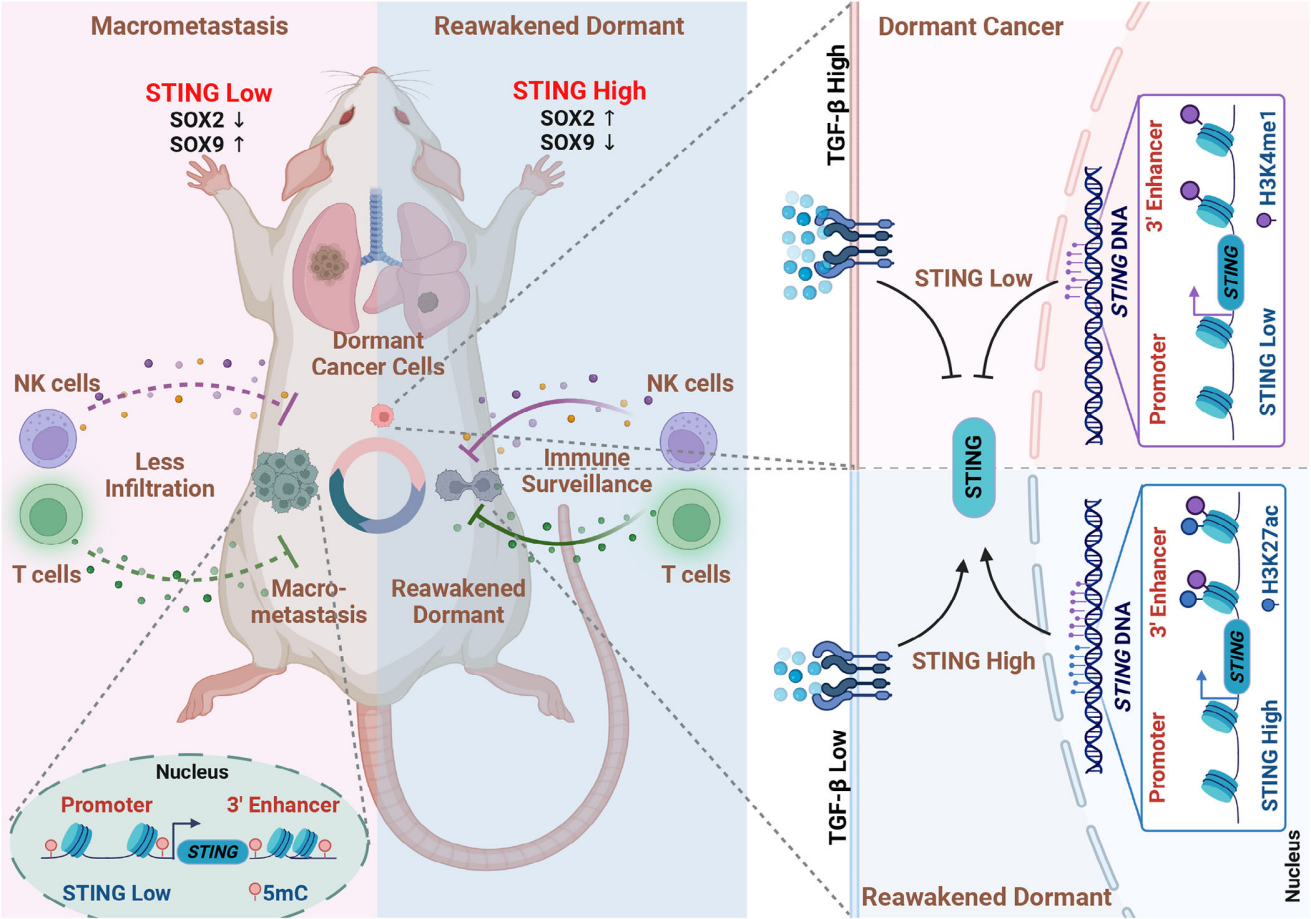
Stimulators of interferon genes (STING) activation prevents the emergence of dormant metastasis in lung adenocarcinoma. The reawakening dormant cancer cells exhibit a relatively high expression of STING, thereby eliciting immune surveillance to attenuate the dormant metastasis. After the spontaneous outbreak of cancer cell macrometastasis, the STING expression level was downregulated. The infiltration of immune cells, such as natural killer (NK) cells and T cells, at the macrometastatic lesion is insufficient to impede the progression of metastasis effectively. In dormant states of cancer cells, the activity of STING is attenuated by transforming growth factor‐β (TGF‐β) induced *STING* enhancer inhibition via loss of the H3K27ac activation, whereas in macrometastatic outbreaks, the expression of STING is suppressed as a result of the *STING* promoter and enhancer hypermethylation.

Immunoregulation has been demonstrated to inhibit the progression of indolent cancer cells toward aggressive metastasis.^3^ To discover the important immune‐related factors involved in dormant metastasis, the authors performed an in vivo CRISPR screen targeting 220 factors that consists natural killer (NK) cell‐activating ligands and major histocompatibility complex class I molecules using human H2087‐LCC and mouse KPad1 LUAD cells. At the endpoint, the metastatic tumor cells were frequently observed in multiple organs. By isolating and analyzing the enrichment of single guide RNA (sgRNA) in metastatic cells from the tumor invaded tissues, the STING signaling‐related genes and its downstream genes, such as *STING*, *IFNB1*, *CCL*, *IFI27*, and *IFITM3*, were identified as the most involved hits in both screens.

To uncover the relationship between STING activation and dormancy awakening, the expressions of STING and Ki67 (a proliferation maker) were analyzed in both dormant single cancer cell niches and macrometastatic lesions in H2087‐LCC cells bearing mice models. Both STING and Ki67 were rarely expressed in dormant cells from isolated lung organs. Interestingly, the awakening dormant cells with high proliferation tendency (Ki67^+^) were found exclusively high STING expression. However, in the outbreak states of cancer cells, the STING was relatively low expressed similar to those of dormant cells. To figure out if the tendency observed in mice models was consistent in human, metastasis and primary tumors from the same LUAD patients were studied. The macrometastatic regions showed significantly diminished STING activation in comparison to the primary tumor site in patients, which indicated the similar LUAD development process between mice and humans. These findings highlight the critical function of STING in mediating the transition process of cancer cells from dormancy to metastasis in LUAD.

To investigate how the dormant and outbreak cells regulate cancer progression through the immune system by controlling the STING activity, the authors initially studied the correlation between STING and immune monitoring‐related factors in different states of cancer cells (e.g., SOX2^+^ progenitors that are more likely to be suppressed by NK cells, and SOX9^+^ cells that exhibit resistance to NK cell‐mediated clearance).^4^ The awakening dormant cancer cells were presented by the cultured isolated dormant cancer cells from H2087‐LCC bearing mice model. In order to compare not only SOX2/SOX9 expression level changes, but also the STING and its related genes activation statues, the single cell RNA sequencing datasets of the reawakened dormant and macrometastatic cancer cells were analyzed. The reawakened dormant cells have relatively high levels of SOX2 and STING, while the macrometastatic cells exhibit increased SOX9 and decreased STING expression. At the same time, the high STING expression in awakening dormant cells were companied with higher interferon‐α (IFNα) signaling pathway activation than macrometastatic cells. The results demonstrate that low STING expression is highly correlated with dormant metastasis development of cancer cells escaping from immune surveillance.

Next, gene editing was performed on H2087‐LCC cells and intracardially inoculated in mice to elucidate the role of STING in the process of metastasis progression in vivo. After 70 days, the STING knockout cell bearing mice showed significantly lower metastasis‐free survival rate compared to the mice with STING wild‐type tumor cells. In contrast, the athymic mice bearing STING overexpressed tumor cells showed higher metastasis‐free survival rate compared to the mice carrying wild‐type tumor cells. These results can be explained by the overexpression of STING, which activates both innate and adaptive immune system, thereby suppressing the outbreak of awakening dormant tumor cells. Interestingly, knockout or decrease activity of STING in tumor cells is unable to influence the direct function of immune cells, but can restrict the NK cells and T cells infiltrating into the burst tumor sites. Researchers furtherly identified two possible factors that contribute to low intrinsic STING expression in dormant and macrometastatic cancer cells. On the one hand, highly secreted transforming growth factor‐β (TGF‐β) promotes dormancy by repressing the *STING* enhancer to downregulate its expression in dormant cells. On the other hand, DNA hypermethylation of the *STING* promoter and enhancer suppresses STING expression in macrometastatic outbreaks.

As STING can sense the cytosolic DNA to activate the innate immune system by inducing type I IFNs and inflammatory cytokines, the STING modulators have been evaluated in clinical trials for treating advanced solid tumor, inflammatory diseases, and autoimmune diseases. In this study, unlike the conventional drug testing in cell line‐derived xenograft or patient‐derived xenograft mice models for assessing tumor size reduction, the authors established mice model harboring DTCs to investigate the dormant metastasis and disseminated tumor entities (DTEs). The mice bearing DTCs (KPad1 or H2087‐LCC) were subjected to the treatment with STING agonist benzothiophene oxobutanoic acid (MSA‐2). The administration of MSA‐2 resulted in a reduction of the DTEs occurrence and displayed an improvement in both metastasis‐free and overall survival rates among mice with normal STING expression cancer cells. However, this observed MSA‐2 effect was not significant in STING knock out tumor cells bearing mice, indicating the suppression of metastasis incidence is dependent on the presence of STING in cancer cells.

Collectively, Hu et al. provide a novel therapeutic perspective on STING activation, which has the potential for preventing the dormant metastasis and reducing the risk of unexpected macrometastasis outbreak following primary therapy. The current STING agonists in clinical settings remain limited in terms of their capacity to enhance innate immune responses and induce apoptosis in cancer cells.^5^ These multifaceted effects may complicate the clinical application of STING agonists in established tumors. Moreover, the immune microenvironment of the dormant metastatic is quite different from the macrometastatic lesions. The findings of this study highlight the importance for researchers to exploit the specific vulnerabilities of dormant metastasis, strategically harnessing the immune system by activating STING activity as an adjuvant therapy to prevent macrometastasis in the foreseeable future for LUAD patients.

## AUTHOR CONTRIBUTIONS

J.M. and X.L. contributed equally to this work. J.M. and X.L. conceived and drafted the manuscript. J.M. drew the figure. S.L. provided valuable discussion and revised the manuscript and figure. All authors have read and approved the article.

## CONFLICT OF INTEREST STATEMENT

The authors declare they have no conflicts of interest.

## ETHICS STATEMENT

Not applicable.

## Data Availability

Not applicable.
